# Development of Agonist-Based PROTACs Targeting Liver X Receptor

**DOI:** 10.3389/fchem.2021.674967

**Published:** 2021-05-26

**Authors:** Hanqiao Xu, Nobumichi Ohoka, Hidetomo Yokoo, Kanako Nemoto, Takashi Ohtsuki, Hiroshi Matsufuji, Mikihiko Naito, Takao Inoue, Genichiro Tsuji, Yosuke Demizu

**Affiliations:** ^1^ Division of Organic Chemistry, National Institute of Health Sciences, Kanagawa, Japan; ^2^ Food Bioscience and Biotechnology, College of Bioresource Sciences, Nihon University, Fujisawa, Japan; ^3^ Division of Molecular Target and Gene Therapy Products, National Institute of Health Sciences, Kanagawa, Japan; ^4^ Laboratory of Targeted Protein Degradation, Graduate School of Pharmaceutical Sciences, The University of Tokyo, Tokyo, Japan; ^5^ Graduate School of Medical Life Science, Yokohama City University, Kanagawa, Japan

**Keywords:** liver X receptor, PROTAC, ubiquitin-proteasome system, von Hippel-Lindau, protein degradation

## Abstract

Liver X receptors (LXRs) belong to the nuclear hormone receptor superfamily and function as ligand-dependent transcription factors that regulate cholesterol homeostasis, lipid homeostasis, and immune responses. LXR antagonists are promising treatments for hypercholesterolemia and diabetes. However, effective LXR antagonists and inhibitors are yet to be developed. Thus, we aimed to develop LXR degraders (proteolysis targeting chimeras PROTACs against LXR) as a complementary strategy to provide a similar effect to LXR inhibition. In this study, we report the development of GW3965-PEG5-VH032 (**3**), a PROTAC capable of effectively degrading LXRβ protein. Compound **3** induced the ubiquitin-proteasome system-dependent degradation of the LXRβ protein, which requires VHL E3 ligase. We hope that PROTACs targeting LXR proteins will become novel therapeutic agents for LXR-related diseases.

## Introduction

Liver X receptor (LXR) is a ligand-dependent transcription factor belonging to the nuclear hormone receptor superfamily (Edwards et al., 2002). Two isoforms, LXRα and LXRβ, have high amino acid sequence homology (78%) but different expression distributions. LXRα is mainly expressed in the liver, intestines, macrophages, and kidneys, whereas LXRβ is ubiquitously expressed in various tissues (Zhu and Li, 2009). Ligand-unbound LXR forms a repressor complex at the LXR target gene promoter. When the ligand binds, it dissociates from the corepressor complex and recruits coactivators such as thyroid hormone receptor-associated protein (TRAP220/DRIP-2) to the target promoter (Wagner et al., 2003; Phelan et al., 2008). LXRs play a pivotal role in the transcriptional regulation of cholesterol homeostasis, fatty acid metabolism, glycolysis, immune responses, and inflammatory responses (Janowski et al., 1996; Wang and Tontonoz, 2018).

Oxidized cholesterol derivatives (oxysterols) such as (22*R*)-22-hydroxycholesterol, (20*S*)-22-hydroxycholesterol, and (24*S*)-24,25-epoxycholesterol are known to be endogenous ligands for LXR, and they activate both LXRα and LXRβ (Baranowski, 2008). In addition, a variety of synthetic LXR ligands have been reported, including isoform-selective ligands (Kick et al., 2016; Kirchgessner et al., 2016). LXR agonists have potential applications as cholesterol-lowering drugs and treatments for atherosclerosis. However, their clinical use is limited because they promote hepatic steatosis by increasing hepatic lipid synthesis (Grefhorst et al., 2002; Viennois et al., 2012). On the other hand, inverse agonists (Griffett et al., 2013; Flaveny et al., 2015) and antagonists (Noguchi-Yachide et al., 2009; Moriwaki et al., 2014; Renga et al., 2015) for LXRs have been developed because inhibiting excessive activation of LXR in the liver is an appropriate strategy to improve hepatic lipid metabolism. However, they have issues with their weak activity and difficulty in chemical synthesis. Thus, the development of complementary strategies could help realize the full potential of LXR inhibition.

Proteolysis targeting chimera (PROTAC) technology has been receiving much attention as a novel strategy to degrade proteins of interest (POI) (Pavia and Crews, 2019; Sun et al., 2019). PROTACs are bifunctional molecules with a ligand for the POI and a ligand for an E3 ligase. PROTACs cross-link between the POI and E3 ligase, which in turn degrades POI by the ubiquitin-proteasome system (UPS). Hence, PROTACs are expected to be a promising tool for suppressing the function of disease-related proteins in drug discovery. Therefore, we hypothesized that we could produce promising compounds with LXR inhibitory activity by developing PROTACs using reported agonists (Gustafson et al., 2015). Herein, we report the design and synthesis of LXR-agonist-based PROTACs which exhibit LXR degradation activity via the UPS.

## Result and Discussion

**FIGURE 1:**
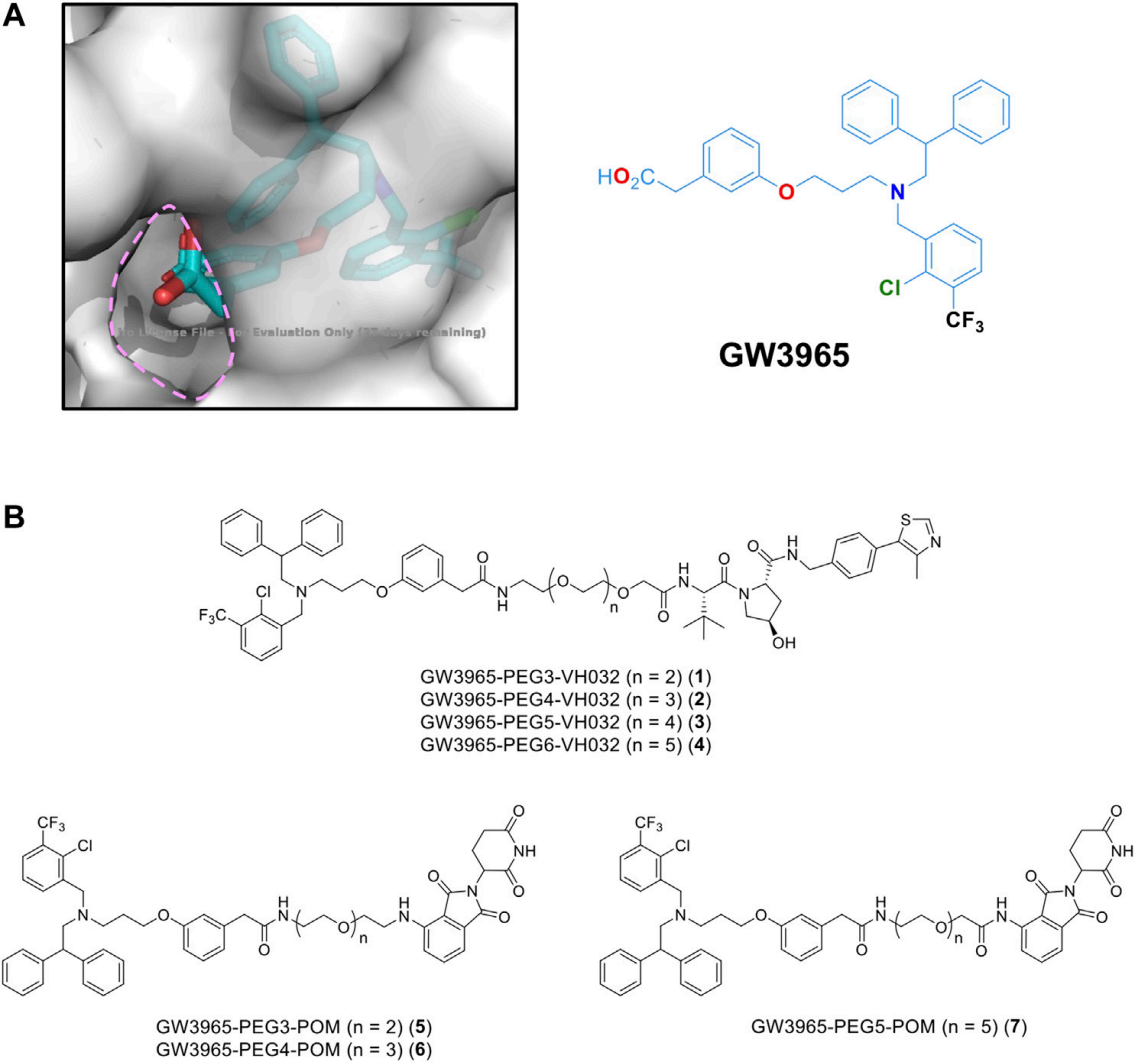
**(A)** X-ray crystal structure of a GW3965-LXRα complex (PDB: 3IPQ). **(B)** Designated PROTACs in this study.

For PROTAC design, a potent LXRα/LXRβ agonist GW3965 (Collins et al., 2002) was selected because the binding mode between LXRα and GW3965 has previously been determined by X-ray diffraction (Fradera et al., 2010). In the co-crystal structure, the carboxyl group of GW3965 is oriented toward the outside of the protein (Figure 1A). Based on this structural information, we designed a GW3965-based PROTAC by linking the carboxyl group with an E3 ligase ligand via a polyethylene glycol linker (PEG3–PEG6). Two representative types of E3 ligase ligands, pomalidomide binding to cereblon (CRBN) and VH032 binding to Von Hippel-Lindau (VHL), were selected (Figure 1B).

**SCHEME 1:**
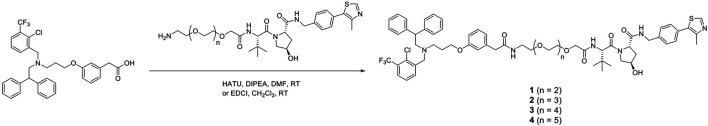
Synthesis of PROTACs for LXR (GW3965-PEG-VH032, **1**–**4**).

The representative synthetic route for VH032-based PROTACs, GW3965-(PEG3–PEG6)-VH032, **1**–**4** is shown in Scheme 1. Ligand GW3965 was conjugated with E3 ligase ligand VH032 with PEG linkers of different lengths via a condensation reaction using HATU/DIPEA or EDCI. Other molecules, including pomalidomide-based PROTAC, were also synthesized in a similar manner, as shown in the Supplementary Material.

**FIGURE 2:**
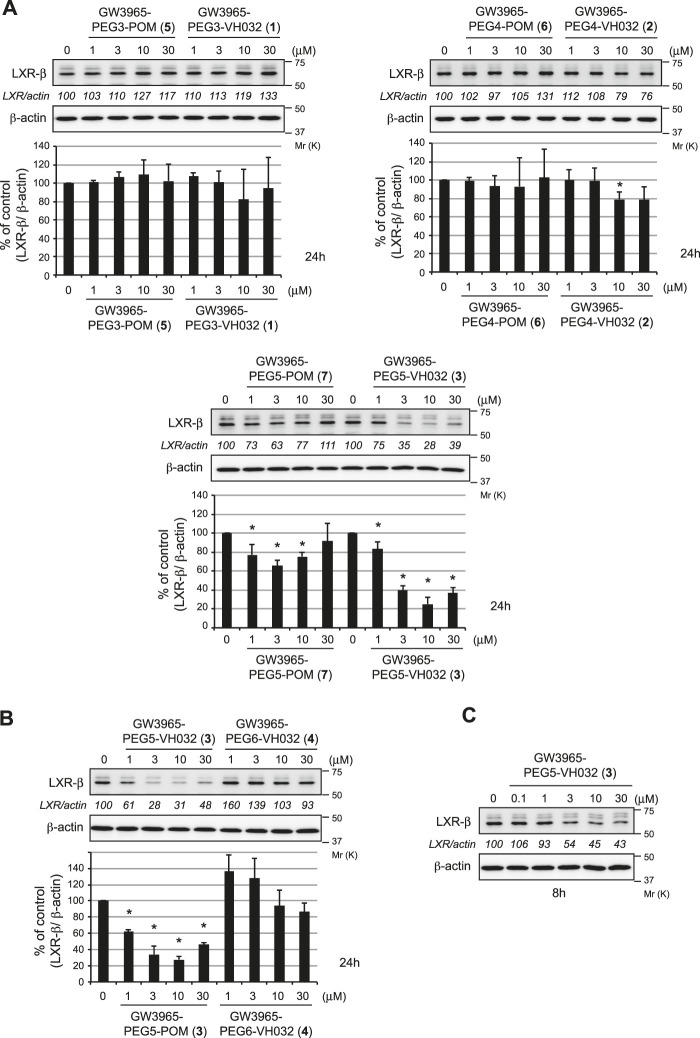
Degradation of the LXRβ protein by the synthesized compounds. HuH-7 cells that had been cultured in Dulbecco’s modified Eagle’s medium (DMEM) containing 10% fetal bovine serum (FBS) were treated with the indicated compounds for 24 h **(A, B)** or 8 h **(C)**. Immunoblots of the cell lysates that had been stained with the indicated antibodies are shown (representative data are shown). The numbers below the LXRβ panels represent LXR/actin normalized by designating the expression from the vehicle control condition as 100%. Data in the bar graph are the mean ± S.D. (error bars) of three independent experiments. Asterisks indicate *p* < 0.05 compared with vehicle control.

The degradation activities of the synthesized chimeric compounds against target proteins, LXRα and LXRβ, that bind to GW3965 were evaluated by western blot using HuH-7 human hepatoma cells expressing the target proteins. Since we could not obtain the appropriate antibodies to detect endogenous LXRα, only the results for LXRβ are shown. We first evaluated the LXRβ reduction activities of a series of chimeric compounds containing different E3 ligands (pomalidomide for CRBN and VH032 for VHL) or different linker lengths (PEG3, PEG4, and PEG5). Compound **3** showed the most potent activity among them (Figure 2A and Supplementary Figure S8). To investigate the optimal linker length in the VHL series, compound **4** with PEG6 linker was synthesized. The reduction activity was almost lost with this linker extension, suggesting that the PEG5 length is optimal (Figure 2B). Compound **3** effectively reduced LXRβ protein levels even after 8 h (Figure 2C). The LXRβ binding affinity (EC_50_) of compound **3** was determined using a time-resolved fluorescence energy transfer (TR-FRET) assay with GW3965 as a positive control. This confirmed that the EC_50_ values of compound **3** (EC_50_ = 31 ± 4.4 nM) were comparable to that of GW3965 (EC_50_ = 20 ± 7.2 nM) (Table 1). As observed in the results of compound **3** and **7** (Figure 2A), the protein degradation efficacy by PROTAC molecules was often suppressed at higher concentrations, which is known as a hook effect (Bondeson et al., 2015). This effect is explained by the inhibition of ternary complex formation (E3-PROTAC-target) by an excess amount of bivalent compounds such as PROTACs.

**TABLE 1:** Binding affinities (EC_50_; half maximal effective concentration) of compounds against LXRβ determined by TR-FRET coactivator assays.

Compounds	EC_50_ (nM)
GW3965	20 ± 7.2
Compound **3**	31 ± 4.4

**FIGURE 3:**
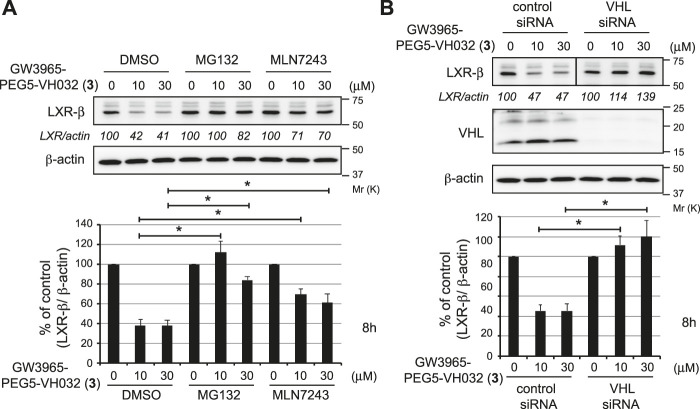
Mechanism of LXRβ reduction by GW3965-PEG5-VH032 (**3**). **(A)** Effect of UPS inhibitors on GW3965-PEG5-VH032-induced reduction of the LXRβ protein. HuH-7 cells that had been cultured in DMEM containing 10% FBS were treated with the indicated concentrations of GW3965-PEG5-VH032 in the presence or absence of 10 μm of MG132 or MLN7243 for 8 h **(B)** VHL E3 ligase is required for the degradation of the LXRβ protein by GW3965-PEG5-VH032. HuH-7 cells were transfected with the VHL siRNA for 42 h and treated with the indicated concentrations of GW3965-PEG5-VH032 for 8 h. A mixture of three different siRNAs against VHL was used to suppress expression. Immunoblots of cell lysates that had been stained with the indicated antibodies are shown (representative data are shown). The numbers below the LXRβ panels represent LXR/actin normalized by designating the expression from the vehicle control condition as 100%. Data in the bar graph are the mean ± S.D. (error bars) of three independent experiments. Asterisks indicate *p* < 0.05.

To investigate the mechanism of LXRβ reduction by compound **3**, we examined the effect of UPS inhibitors (Figure 3A). Compound **3**-induced decrease in the LXRβ protein was abrogated by co-treatment with a proteasome inhibitor, MG132, and a ubiquitin-activating inhibitor, MLN7243, indicating that the compound induces UPS-dependent degradation of the LXRβ protein. To confirm whether VHL is required for the degradation of the LXRβ protein by compound **3**, we examined the effect of silencing the E3 ligase by short interfering RNA (siRNA) (Figure 3B). The depletion of VHL by siRNAs completely suppressed the degradation of the LXRβ protein by compound **3**, indicating that VHL is required for degradation.

## Conclusion

Herein, we report the synthesis of a PROTAC for LXR degradation as an effective inhibitory molecule. In the molecular design, the linking position of chimeric compounds was determined based on the structural information from X-ray crystallography of LXRα and its agonist GW3965. For the E3 ligase ligand in the PROTAC, VH032 and pomalidomide were introduced into chimeric compounds. The LXRβ degradation activity of the synthesized PROTACs was evaluated by western blot using HuH-7 human hepatoma cells, and it was found that the activity of VH032-based PROTACs (GW3965-PEG-VH032) was more potent than that of pomalidomide-based PROTACs (GW3965-PEG-POM) between the PEG3-PEG5 linkers. To investigate the effect of the linker length on the degradation activity, a series of VH032-type PROTACs with PEG3–PEG6 were examined, which revealed that the PROTAC with PEG5 (GW3965-PEG5-VH032, **3**) exhibits the most potent activity for LXRβ degradation among them. Compound **3** was confirmed to bind to LXRβ, inducing its degradation. LXRβ degradation by this molecule occurs via the ubiquitin-proteasome system mediated by VHL E3 ligase. The degraders developed in this study have potential as novel therapeutic agents for LXR-related diseases. Therefore, our results suggest that agonist-based PROTACs could be a new approach to create PROTACs, even in the absence of an appropriate antagonist as a binding ligand for the POI.

## Data Availability

The original contributions presented in the study are included in the article/Supplementary Material, further inquiries can be directed to the corresponding authors.
