# Supplementary material for: Development of Agonist-Based PROTACs Targeting Liver X Receptor
Source: Front Chem. 2021 May 26;9:674967. doi: 10.3389/fchem.2021.674967 (PMC8187946; doi:10.3389/fchem.2021.674967)
Supplement: Supplementary file 1 [file DataSheet1.docx]

Supplementary Material for

Development of agonist-based PROTACs targeting Liver X Receptor

# Supplementary Data

Experimental details and spectroscopic data for new compounds are provided in this Supplementary Material.

**General**

All chemicals were purchased from Sigma-Aldrich Co. LLC, Kanto Chemicals Co. Inc., Tokyo Chemical Industry Co. Ltd., Wako Pure Chemical Industries Ltd., and were used without further purification. Reactions were followed by thin-layer chromatography (TLC) (60 F254, Merck), and spots were visualized by UV irradiation with a handheld UV lamp (254/365 nm) (UVP) and iodine vapor or ninhydrin reagent. Silica gel for column chromatography was Kanto Chemical 60N (spherical, neutral), NH silica gel (Chromatrex NH-DM1020, Fuji Silicia), or packed columns for medium pressure column chromatography (Hi-Flash column / Inject column Yamazen). ^1^H and ^13^C NMR spectra were measured on an ECZ 600R spectrometer (JEOL) using deuterated solvents. Chemical shift values (ppm) were corrected for residual solvent signals as internal standards (CD_3_OD: 3.30 for ^1^H NMR, 49.0 for ^13^C NMR). The splitting modes of the signals are as follows (singlet (s), doublet (d), triplet (t), quartet (q), double of doublets (dd), multiplet (m), broad (br)). High-resolution mass spectrometry (HRMS) was measured by electrospray ionization using Shimadzu IT-TOF MS (Shimadzu).

Compounds **8**^1)^, **10**^1)^, **16**^2)^ and **18**^3)^ were prepared according to reported methods. **12**, **14**, and **20** were purchased from Sigma-Aldrich.

**Supplementary Scheme 1.** Synthesis of LXR-PROTACs **1**-**7**.

**(2*S*,4*R*)-1-((*S*)-14-amino-2-(*tert*-butyl)-4-oxo-6,9,12-trioxa-3-azatetradecanoyl)-4-hydroxy-*N*-(4-(4-methylthiazol-5-yl) benzyl) pyrrolidine-2-carboxamide (9)**

**8** (40 mg, 0.062 mmol) was dissolved in methanol (1.5 mL), and then 10% Pd/C (10 mg) was added. The reaction mixture was stirred for 2 hours while exchanging hydrogen gas. The reaction solution was filtered through a pad of Celite, concentrated under reduced pressure, and dried in vacuo to give **9**. ESI-HRMS calcd for C_30_H_46_N_5_O_7_S [M+H]^2+^: 310.1556, found: 310.6493.

**(2*S*,4*R*)-1-((*S*)-2-(*tert*-Butyl)-17-(3-(3-((2-chloro-3-(trifluoromethyl) benzyl) (2,2-diphenylethyl) amino) propoxy) phenyl)-4,16-dioxo-6,9,12-trioxa-3,15-diazaheptadecanoyl)-4-hydroxy-*N*-(4-(4-methylthiazol-5-yl) benzyl) pyrrolidine-2-carboxamide (1)**

A mixture of **9** (40 mg, 0.065 mmol), GW3965 (52 mg, 0.095 mmol), HATU (62 mg, 0.16 mmol), *N*, *N*-diisopropylethylamine (52 μL, 0.33 mmol) in *N*, *N*-dimethylformamide (650 μL) was stirred with the mixture was stirred at room temperature for 3 hours. The reaction solution was concentrated under reduced pressure and purified by HPLC (gradient: 30-100% MeCN-H_2_O containing 0.1% TFA, 20 min) to give **1** in 32% (24.5 mg) yield.; ^1^H NMR (δ, ppm, 600 MHz, CD_3_OD): δ = 8.98 (s, 1H), 7.90 (d, *J* = 7.8 Hz, 1H), 7.78 (d, *J* = 7.2 Hz, 1H), 7.55 (t, *J* = 7.8 Hz, 1H), 7.43-7.37 (m, 4H), 7.34-7.29 (m, 8H), 7.23 (t, *J* = 7.2 Hz, 2H), 7.16 (t, *J* = 7.8 Hz, 1H), 6.86 (d, *J* = 7.8 Hz, 1H), 6.73 (s, 1H), 6.64 (dd, *J* = 8.4, 2.4 Hz, 1H), 4.73 (br, 2H), 4.67-4.65 (m, 1H), 4.60 (t, *J* = 7.8 Hz, 1H), 4.55-4.52 (m, 1H), 4.46-4.44 (m, 1H), 4.33-4.30 (m, 1H), 4.08 (br, 2H), 4.02-3.93 (m, 5H), 3.83-3.74 (m, 2H), 3.65-3.54 (m, 8H), 3.51-3.43 (m, 6H), 3.32-3.29 (m, 4H), 2.44 (s, 3H), 2.27 (br, 2H), 2.20-2.17 (m, 1H), 2.06-2.02 (m, 1H), 1.00 (s, 9H). ^13^C NMR (δ, ppm, 151 MHz, CD_3_OD): δ = 173.0, 172.5, 170.7, 170.3, 159.6, 159.4, 158.3, 152.0, 146.8, 139.9, 139.2, 137.3, 137.1, 133.3, 129.3, 129.1, 128.1, 127.7, 127.6, 127.5, 121.9, 115.3, 112.8, 70.8, 70.3, 70.2, 69.9, 69.2, 64.9, 59.5, 57.9, 56.8, 56.8, 55.1, 53.8, 52.5, 48.5, 48.1, 47.9, 47.8, 47.7, 47.5, 47.4, 47.2, 46.8, 42.4, 42.3, 39.3, 37.7, 35.8, 25.6, 22.9, 14.10. ESI-HRMS calcd for C_63_H_76_ClF_3_N_6_O_9_S [M+H]^2+^: 592.2512, found: 592.2520. Purity 98% (Rt = 18.37 min).

**(2*S*,4*R*)-1-((*S*)-17-amino-2-(*tert*-butyl)-4-oxo-6,9,12,15-tetraoxa-3-azaheptadecanoyl)-4-hydroxy-*N*-(4-(4-methylthiazol-5-yl) benzyl) pyrrolidine-2-carboxamide (11)**

Employing the procedure described above for **9** and starting from compound **10** afforded compound **13**; ^1^H NMR (δ, ppm, 600 MHz, CD_3_OD): δ = 8.88 (s, 1H), 7.46 (d, *J* = 8.1 Hz, 2H), 7.42 (d, *J* = 8.1 Hz, 2H), 4.67 (s, 1H), 4.58-4.51 (m, 3H), 4.35 (s, 1H), 4.08-4.05 (m, 2H), 3.90-3.88 (m, 1H), 3.81 (dd, *J* = 10.8, 4.2 Hz, 1H), 3.72-3.58 (m, 18H), 2.96 (t, *J* = 4.8 Hz, 2H), 2.48 (s, 3H), 2.24-2.22 (m, 1H), 2.11-2.07 (m, 1H), 1.26 (s, 1H), 1.02 (s, 9H). ESI-HRMS calcd for C_32_H_50_N_5_O_8_S [M+H]^2+^: 332.1688, found: 332.6685.

**(2*S*,4*R*)-1-((*S*)-2-(*tert*-Butyl)-20-(3-(3-((2-chloro-3-(trifluoromethyl) benzyl) (2,2-diphenylethyl) amino) propoxy) phenyl) -4,19-dioxo-6,9,12,15-tetraoxa-3,18-diazaicosanoyl)-4-hydroxy-*N*-(4-(4-methylthiazol-5-yl) benzyl) pyrrolidine-2- carboxamide (2)**

A similar procedure to that of **1** was employed for the preparation of compound **2**.

^1^H NMR (δ, ppm, 600 MHz, CD_3_OD): δ = 8.90 (s, 1H), 7.89 (d, *J* = 7.8 Hz, 1H), 7.75 (d, *J* = 7.2 Hz, 1H), 7.54-7.52 (m, 1H), 7.41 (d, *J* = 7.8 Hz, 2H), 7.37 (d, *J* = 7.8 Hz, 2H), 7.30-7.28 (m, 8H), 7.22 (t, *J* = 7.2 Hz, 2H), 7.15 (t, *J* = 7.2 Hz, 1H), 6.85 (d, *J* = 7.2 Hz, 1H), 6.72 (s, 1H), 6.64-6.62 (m, 1H), 4.71-4.70 (m, 1H), 4.65 (d, *J* = 9.6 Hz, 1H) 4.58 (d, *J* = 6.6 Hz, 1H), 4.53-4.43 (m, 3H), 4.33-4.30 (m, 1H), 4.06-3.93 (m, 6H), 3.82-3.73 (m, 2H), 3.65-3.57 (m, 9H), 3.55-3.53 (m, 2H), 3.51-3.50 (m, 2H), 3.46 (d, *J* = 5.4 Hz, 2H), 3.43-3.42 (m, 3H), 3.18-3.13 (m, 1H), 2.43 (s, 3H), 2.56 (br, 2H), 2.19-2.16 (m, 1H), 2.09-1.98 (m, 1H), 1.89 (s, 1H), 1.36-1.22 (m, 2H), 1.01 (s, 9H). ^13^C NMR (δ, ppm, 151 MHz, CD_3_OD): δ = 173.0, 172.5, 170.7, 170.3, 159.6, 159.3, 158.3, 151.8, 147.2, 139.9, 139.1, 137.3, 137.1, 133.3, 132.4, 129.9, 129.7, 129.3, 129.3, 129.1, 128.1, 127.6, 127.5, 121.9, 121.8, 115.3, 112.8, 70.9, 70.3, 70.2, 70.2, 69.9, 69.8, 69.7, 69.1, 64.9, 59.5, 57.9, 56.7, 56.6, 55.12, 52.5, 48.5, 48.2, 48.1, 47.9, 47.8, 47.7, 47.5, 47.4, 47.2, 46.8, 46.6, 42.4, 42.3, 39.3, 37.7, 35.8, 25.6, 23.0, 14.3, 7.9. ESI-HRMS calcd for C_65_H_80_ClF_3_N_6_O_10_ [M+H]^2+^: 614.2643, found: 614.2648. Purity 96% (Rt = 18.37 min).

**(2*S*,4*R*)-1-((*S*)-2-(*tert*-Butyl)-23-(3-(3-((2-chloro-3-(trifluoromethyl) benzyl) (2,2-diphenylethyl) amino) propoxy) phenyl)-4,22-dioxo-6,9,12,15,18-pentaoxa-3,21-diazatricosanoyl)-4-hydroxy-*N*-(4-(4-methylthiazol-5-yl) benzyl) pyrrolidine-2-carboxamide (3)**

A similar procedure to that of **1** was employed for the preparation of compound **3** using compound **12**; ^1^H NMR (δ, ppm, 600 MHz, CD_3_OD): δ = 8.98 (s, 1H), 8.59 (t, *J* = 5.4 Hz, 1H), 8.09 (s, 1H), 7.42-7.38 (m, 5H), 7.37-7.15 (m, 6H), 6.80 (d, *J* = 6.6 Hz, 1H), 4.55 (d, *J* = 9.6 Hz, 1H), 4.44-4.34 (m, 3H), 4.24 (dd, *J* = 15.0, 5.4 Hz, 2H), 3.95 (s, 2H), 3.67-3.64 (m, 1H), 3.59-3.46 (m, 20H), 3.39-3.36 (m, 5H), 3.18 (dd, *J* = 11.4, 6.0 Hz, 2H), 2.49 (s, 14H), 2.43 (s, 3H), 2.04 (t, *J* = 12.6 Hz, 1H), 1,90-1.87 (m, 1H), 0.93 (s, 12H). ESI-HRMS calcd for C_67_H_84_ClF_3_N_6_O_11_S [M+H]^2+^: 636.2774, found: 636.2765.

**(2*S*,4*R*)-1-((*S*)-23-Amino-2-(*tert*-butyl)-4-oxo-6,9,12,15,18,21-hexaoxa-3-azatricosanoyl)-4-hydroxy-*N*-(4-(4-methylthiazol-5-yl) benzyl) pyrrolidine-2-carboxamide (15)**

Employing the procedure described above for **9** and starting from compound **14** afforded compound **15**;

^1^H NMR (δ, ppm, 600 MHz, CD_3_OD): δ = 8.85 (s, 1H), 7.43 (d, *J* = 8.4 Hz, 2H), 7.39 (dd, *J* = 7.8, 1.8 Hz, 2H), 4.67 (s, 1H), 4.52-4.44 (m, 4H), 4.32 (d, *J* = 15.6 Hz, 1H), 4.05-4.02 (m, 2H), 3.84 (d, *J* = 11.4 Hz, 1H), 3.78-3.76 (m, 1H), 3.70-3.52 (m, 26H), 2.73 (t, *J* = 5.4 Hz, 1H), 2.44 (s, 2H), 1.29-1.92 (m, 4H), 1.01 (s, 9H). ESI-HRMS calcd for C_36_H_58_N_5_O_10_S [M+H]^2+^: 376.1950, found: 376.7007.

**(2*S*,4*R*)-1-((*S*)-2-(*tert*-Butyl)-26-(3-(3-((2-chloro-3-(trifluoromethyl) benzyl) (2,2-diphenylethyl) amino) propoxy) phenyl)-4,25-dioxo-6,9,12,15,18,21-hexaoxa-3,24-diazahexacosanoyl)-4-hydroxy-*N*-(4-(4-methylthiazol-5-yl) benzyl) pyrrolidine-2-carboxamide (4)**

A similar procedure to that of **1** was employed for the preparation of compound **4**.

^1^H NMR (δ, ppm, 600 MHz, CD_3_OD): δ = 8.88 (s, 1H), 8.67 (t, *J* = 6 Hz, 1H), 7.86 (br, 1H), 7.70 (d, *J* = 9.0 Hz, 1H), 7.47-7.19 (m, 20H), 6.90 (t, *J* = 7.8 Hz, 1H), 6.76 (s, 1H), 6.66 (dd, *J* = 7.8, 6.0 Hz, 1H), 4.70 (d, *J* = 9.6 Hz, 1H), 4.56-4.46 (br, 4H), 4.36-4.33 (br, 1H), 3.98-3.93 (m, 1H), 3.63-3.57 (m, 26H), 3.52 (t, *J* = 6.0 Hz, 1H), 3.49 (s, 2H), 2.47 (s, 3H), 2.28-2.21 (m, 2H), 2.11-2.06 (m, 1H), 1.94-1.76 (m, 7H), 1.31-1.29 (m, 1H), 1.04 (s, 9H). ESI-HRMS calcd for C_69_H_88_ClF_3_N_6_O_12_S [M+H]^2+^: 658.2905, found: 658.2900.

**4-((2-(2-(2-Aminoethoxy) ethoxy) ethyl) amino)-2-(2,6-dioxopiperidin-3-yl) isoindoline-1,3-dione 2,2,2-trifluoroacetate (17)**

Trifluoroacetic acid (2 mL) was added to the solution of **16** (27.3 mg, 0.054 mmol) in dichloromethane (2 mL), and the mixture was stirred at room temperature for 1 hour. The reaction solution was concentrated under reduced pressure, the resulting liquid was dried in vacuo to give **17** in 47% (9.9 mg). This crude product was used in next step without further purification. ESI-MS calcd for C_19_H_25_N_4_O_6_ [M+H]^+^ : 405.1769, found: 405.1685.

**2-(3-(3-((2-Chloro-3-(trifluoromethyl) benzyl) (2,2-diphenylethyl) amino) propoxy) phenyl)-*N*-(2-(2-(2-((2-(2,6-dioxopiperidin-3-yl)-1,3-dioxoisoindolin-4-yl) amino) ethoxy) ethoxy) ethyl) acetamide (5)**

A mixture of **17** (9.9 mg, 0.019 mmol), GW3965 (15.4 mg, 0.025 mmol), HATU (18.2 mg, 0.048 mmol), *N*, *N*-diisopropylethylamine (15.2 μL, 0.095 mmol) in *N*, *N*-dimethylformamide (220 μL) was stirred with the mixture was stirred at room temperature for 3 hours. The reaction solution was concentrated under reduced pressure and purified by HPLC (gradient: 30-100% MeCN-H_2_O containing 0.1% TFA, 20 min) to give **5** in 76% (14.1 mg) yield.; ^1^H NMR (δ, ppm, 600 MHz, CD_3_OD): δ = 7.76 (d, *J* = 7.8 Hz, 1H), 7.63 (d, *J* = 7.8 Hz, 1H), 7.37-7.29 (m, 1H), 7.19-7.16 (m, 9H), 7.10 (t, *J* = 6.6 Hz, 2H), 7.00 (t, *J* = 7.8 Hz, 1H), 6.87 (q, *J* = 9.0 Hz, 2H), 6.72 (d, *J* = 7.2 Hz, 1H), 6.59 (t, *J* = 1.8 Hz, 1H), 6.50-6.47 (m, 1H), 4.57 (br, 2H), 4.45 (t, *J* = 7.2 Hz, 1H), 3.93 (br, 2H), 3.82 (t, *J* = 5.4 Hz, 3H), 3.53 (t, *J* = 5.4 Hz, 2H), 3.47-3.46 (m, 2H), 3.44-3.43 (m, 2H), 3.38 (t, *J* = 6.0 Hz, 2H), 3.21-3.18 (m, 4H), 3.15-3.13 (m, 8H), 2.62-2.43 (m, 3H), 2.12 (m, 2H). ^13^C NMR (δ, ppm, 151 MHz, CD_3_OD): δ = 173.2, 172.4, 170.3, 169.4, 167.9, 158.3, 146.8, 139.9, 137.3, 137.1, 135.9, 133.2, 132.5, 129.7, 129.5, 129.3, 129.3, 129.1, 128.1, 127.6, 127.5, 123.6, 121.9, 121.8, 116.9, 115.3, 112.7, 110.8, 109.9, 70.2, 69.9, 69.2, 69.1, 64.9, 57.9, 55.1, 52.5, 48.2, 48.1, 47.9, 47.8, 47.7, 47.5, 47.4, 47.2, 46.8, 42.3, 41.9, 39.3, 30.8, 23.0, 22.4. ESI-HRMS calcd for C_52_H_55_ClF_3_N_5_O_8_ [M+H]^2+^: 484.6840, found: 484.6848. Purity 100% (Rt = 19.27 min).

**4-((2-(2-(2-(2-Aminoethoxy) ethoxy) ethoxy) ethyl) amino)-2-(2,6-dioxopiperidin-3-yl) isoindoline-1,3-dione 2,2,2-trifluoroacetate (19)**

Employing the procedure described above for **17** and starting from compound **18** afforded compound **19**, This crude product was used in next step without further purification.; ^1^H NMR (δ, ppm, 600 MHz, CD_3_OD): δ = 7.55 (dd, *J* = 8.4, 7.2 Hz, 1H), 7.09 (d, *J* = 8.4 Hz, 1H), 7.06 (d, *J* = 6.6 Hz, 1H), 5.05(q, *J* = 7.2 Hz, 1H), 3.72 (t, *J* = 5.4 Hz, 2H), 3.69-3.66 (m, 8H), 3.64-3.63 (m, 2H), 3.51 (t, *J* = 5.4 Hz, 2H), 3.08 (t, *J* = 6.0 Hz, 2H), 3.05 (s, 1H), 2.91 (s, 1H), 2.85-2.83 (m, 1H), 2.76-2.68 (m, 2H), 2.11-2.06 (m, 2H), 2.07 (s, 1H), 1.28 (s, 1H). ESI-HRMS calcd for C_21_H_29_N_4_O_7_ [M+H]^+^ : 449.2031, found: 449.1960.

**2-(3-(3-((2-Chloro-3-(trifluoromethyl) benzyl) (2,2-diphenylethyl) amino) propoxy) phenyl)-*N*-(2-(2-(2-(2-((2-(2,6-dioxopiperidin-3-yl)-1,3-dioxoisoindolin-4-yl) amino) ethoxy) ethoxy) ethoxy) ethyl) acetamide (6)**

A similar procedure to that of **5** using compound **19** was employed for the preparation of compound **6**; ^1^H NMR (δ, ppm, 600 MHz, CD_3_OD): δ = 7.77 (d, *J* = 6.0 Hz, 1H), 7.63 (s, 1H), 7.41(dd, *J* = 15, 7.8 Hz, 2H), 7.22-7.06 (m, 15H), 6.94 (q, *J* = 7.8 Hz, 2H), 6.78 (d, *J* = 7.2 Hz, 1H), 6.64 (s, 1H), 6.59-6.53 (m, 2H), 4.91 (dd, *J* = 12, 5.4 Hz, 1H), 4.46 (br, 3H), 3.84 (br, 4H), 3.59-3.36 (m, 12H), 3.23-3.20 (m, 5H), 2.69-2.55 (m, 3H), 2.13 (br, 2H), 1.97-1.93 (m, 1H), 1.28-1.18 (m, 1H). ^13^C NMR (δ, ppm, 151 MHz, CD_3_OD): δ = 174.6, 173.8, 173.6, 171.6, 170.7, 169.3, 162.6, 162.4, 159.8, 148.2, 142.1, 138.6, 137.9, 137.2, 133.9, 131.0, 130.7, 130.6, 130.5, 130.3, 129.1, 128.9, 128.6, 125.0, 123.2, 123.1, 122.9, 118.3, 117.0, 116.6, 116.3, 114.1, 113.9, 112.0, 111.3, 71.7, 71.6, 71.6, 71.3, 70.6, 70.4, 43.7, 43.2, 41.8, 41.3, 40.6, 38.2, 35.9, 32.2, 30.7, 24.9, 23.8. ESI-HRMS calcd for C_54_H_59_ClF_3_N_5_O_9_ [M+H]^2+^: 506.6971, found: 506.6965. Purity 98% (Rt = 19.35 min).

**17-(2-(3-(3-((2-Chloro-3-(trifluoromethyl) benzyl) (2,2-diphenylethyl) amino) propoxy) phenyl) acetamido)-*N*-(2-(2,6-dioxopiperidin-3-yl)-1,3-dioxoisoindolin-4-yl)-3,6,9,12,15-pentaoxaheptadecanamide (7)**

The mixture of Pomalidomide-PEG_5_-NH_2_-HCl (**20**)(11.86 mg, 0.20 mmol), GW3965 (12.56 mg, 0.0203 mmol), 1-ethyl-3-(3-dimethylaminopropyl)carbodiimide hydrochloride (5.86 mg, 0.0306 mmol), 1-hydroxybenzotriazole monohydrate (6.46 mg, 0.042 mmol), *N*, *N*-diisopropylethylamine (9 μL, 0.068 mmol) in *N*, *N*-dimethylformamide (2 mL) was stirred overnight. Acetonitrile was added to the reaction solution and filtered. The reaction solution was concentrated under reduced pressure and purified by HPLC (gradient: 30-100% MeCN-H_2_O containing 0.1% TFA, 20 min) to give **7** in 16% (3.3 mg) yield.; ^1^H NMR (δ, ppm, 600 MHz, CD_3_OD): δ = 11.12 (s, 1H), 10.32 (s, 1H), 8.65 (d, *J* = 8.4 Hz, 1H), 8.05 (br, 1H), 7.82 (t, *J* = 7.2 Hz, 1H), 7.59 (d, *J* = 7.2 Hz, 1H), 7.19-7.13 (m, 12H), 6.77 (d, *J* = 6.6 Hz, 3H), 5.12 (dd, *J* = 13.2, 5.4 Hz, 1H), 4.16 (s, 2H), 3.71 (t, *J* = 5.4 Hz, 2H), 3.62 (t, *J* = 4.8 Hz, 2H), 3.50 (t, *J* = 4.8 Hz, 2H), 3.45-3.42 (m, 10H), 3.35-3.33 (m, 4H), 3.15 (dd, *J* = 5.4, 10.8 Hz, 2H), 2.58 (d, *J* = 2.4 Hz, 1H), 2.46 (s, 16H). ESI-HRMS calcd for C_58_H_65_ClF_3_N_5_O_12_ [M+H]^2+^: 557.7130, found: 557.7146. Purity 100% (Rt = 19.05 min).

**HPLC conditions**

Preparative HPLC was performed using a CAPCELL PAK C18 column (5 μm, 10 mm I.D. × 250 mm, OSAKA SODA) at a flow rate of 3.5 mL/min and eluents were detected at 260 nm by a SPD-10AV, SHIMADZU (Kyoto, Japan). Analytical HPLC was performed using an Inertsil WP300 C18 column (5 μm, 4.6 mm I.D. × 250 mm, GL Science Inc.) at a flow rate of 1.0 mL/min and eluents were detected at 260 nm by an EXTREMA, Jasco (Tokyo, Japan).

**1** Purity 98% (Rt = 18.37 min).


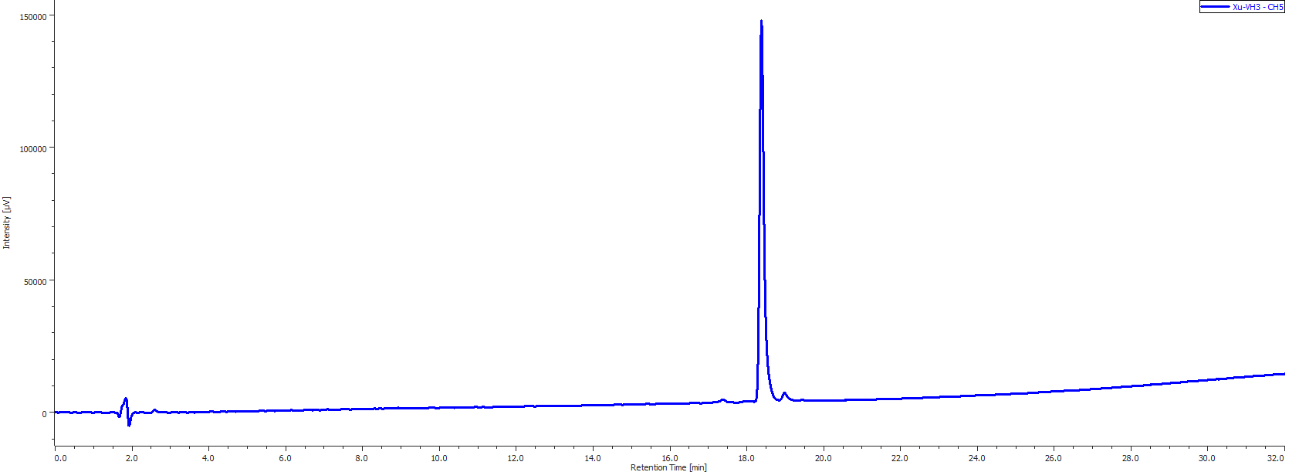


**Supplementary Figure 1.** HPLC trace of compound **1**.

**2** Purity 96% (Rt = 18.37 min).


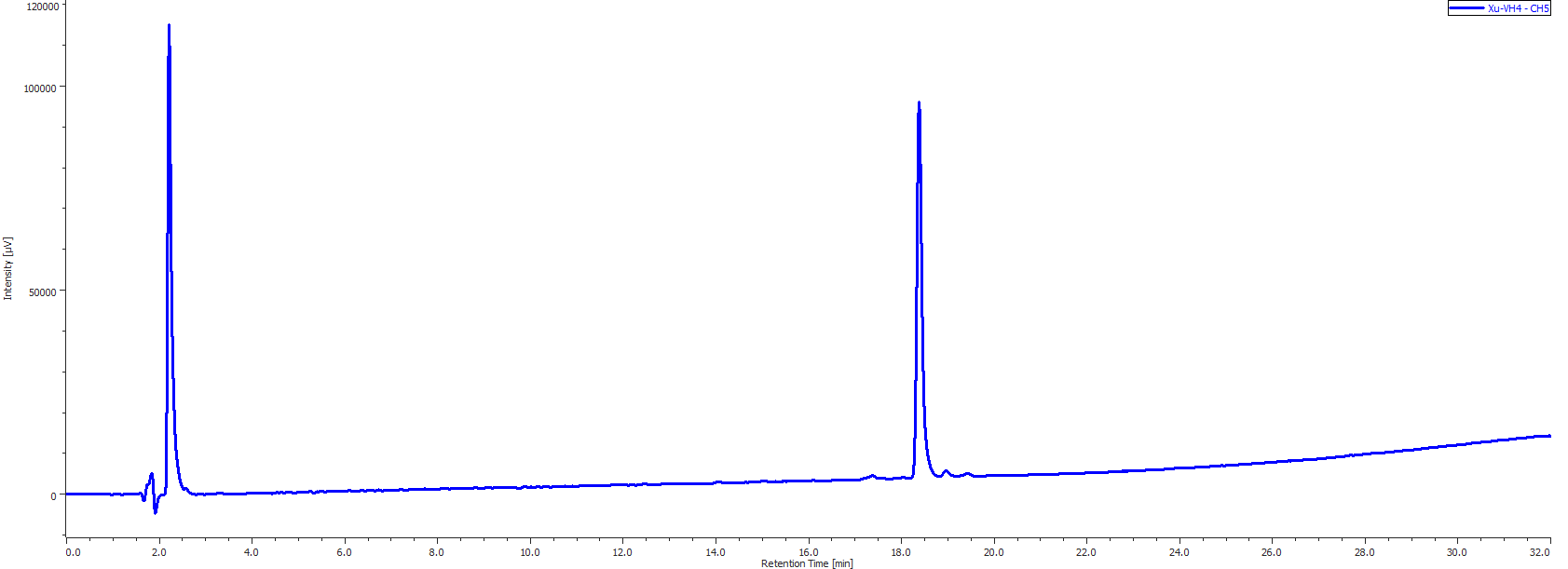


**Supplementary Figure 2.** HPLC trace of compound **2**.

**3** Purity 100% (Rt =18.23 min).


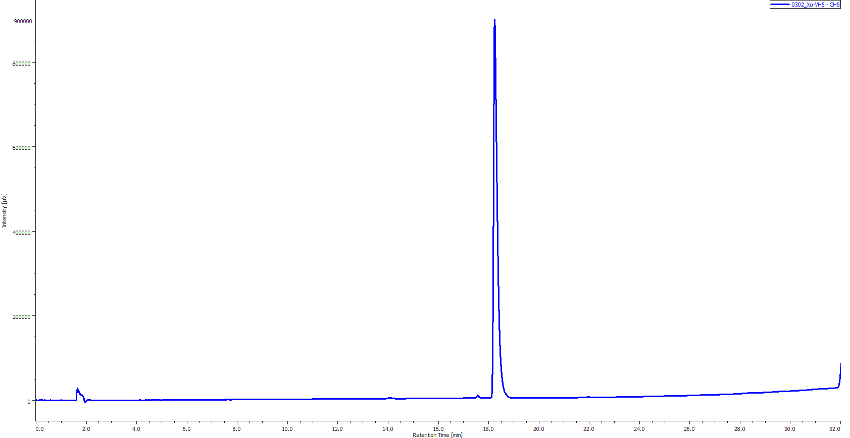


**Supplementary Figure 3.** HPLC trace of compound **3**.

**4** Purity 99% (Rt =18.27 min).


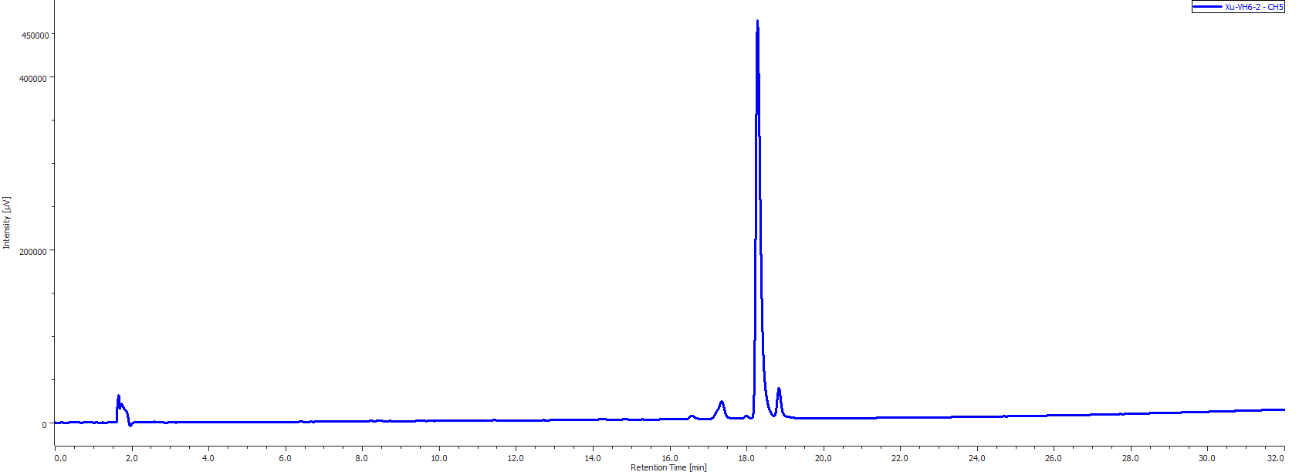


**Supplementary Figure 4.** HPLC trace of compound **4**.

**5** Purity 100% (Rt = 19.27 min)


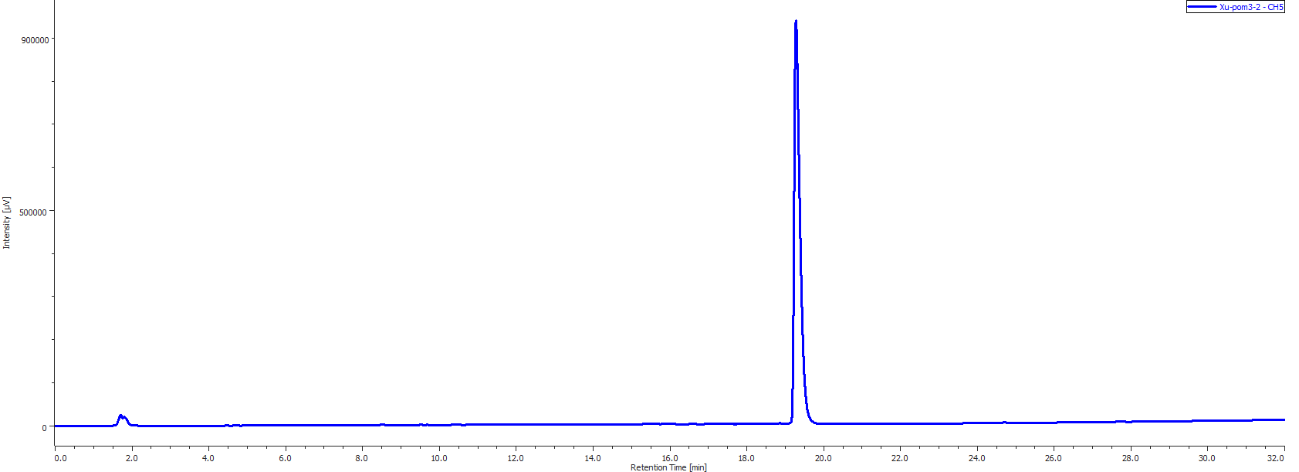


**Supplementary Figure 5.** HPLC trace of compound **5**.

**6** Purity 98% (Rt = 19.35 min)


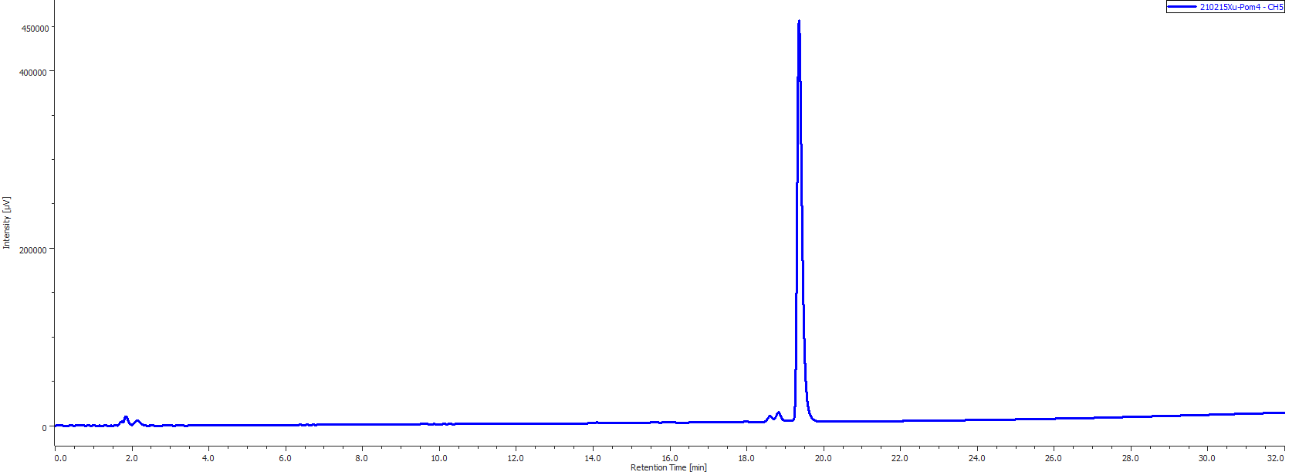


**Supplementary Figure 6.** HPLC trace of compound **6**.

**7** Purity 100% (Rt = 19.05 min)


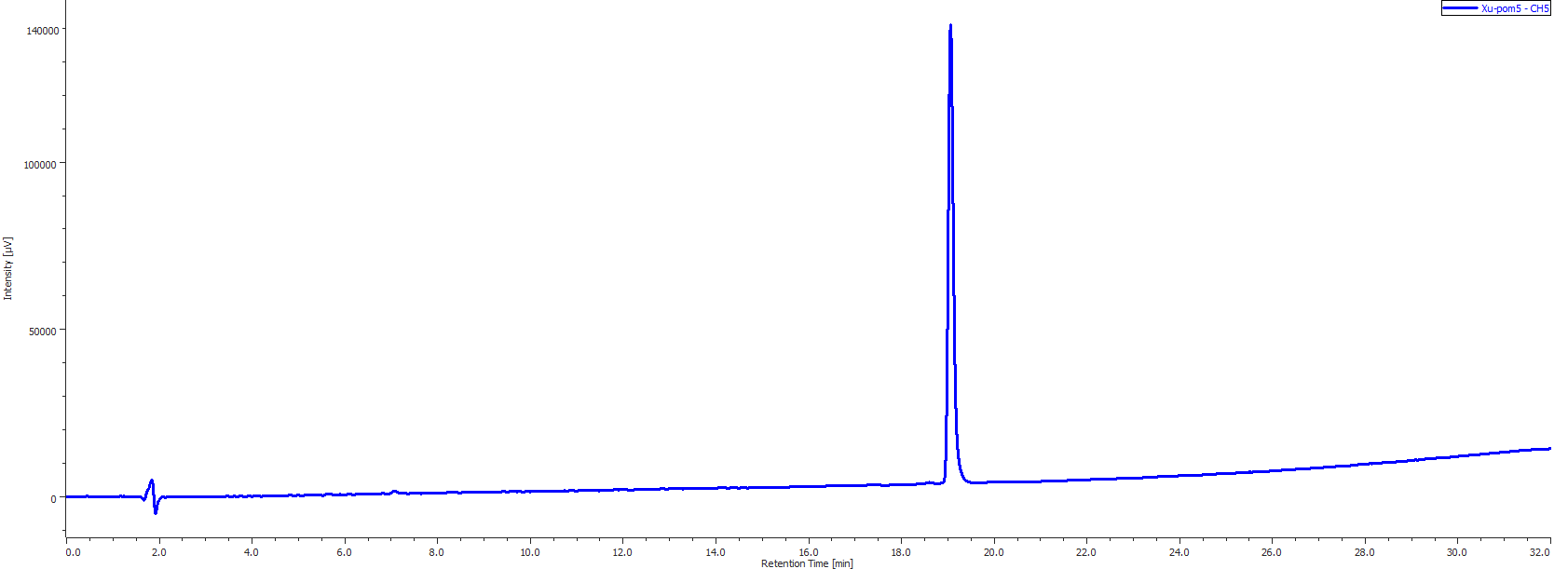


**Supplementary Figure 7.** HPLC trace of compound **7**.

**Supplementary Figure 8.** Comparative analysis of all compounds at same concentrations by Western blotting.


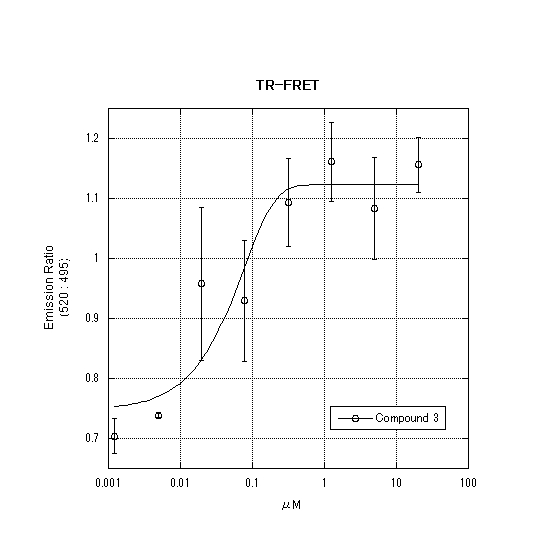


**Supplementary Figure 9.** Curve fitting in TR-FRET LXRβ-Coactivator assay.

Serial dilution of compound **3** (1% final DMSO concentration; 1.2 nM-20 μM)

**Binding assay**

Binding affinity (EC_50_ value) against LXRβ was determined using LanthaScreen® TR-FRET LXR (liver X receptor) beta Coactivator Assay (ThermoFisher Scientific, Waltham, MA, USA; PV4658) according to manufacture’s protocol. This assay was performed using a black, 384-well assay plate (polystyrene, F-bottom, non-binding)(Greiner bio-one, Kremsmünster, Austria), and a fluorescence plate reader, Envision (PerkinElmer, Waltham, MA, USA) was used to detect the fluorescent emission signals of terbium at 495 nm and fluorescein at 520 nm. In these quantitate assay, at least 3 independent experiments were repeated, and the calculated EC_50_ values were represented as their averaged values with standard deviation.

**Western blot analysis**

Cells were lysed with SDS lysis buffer (0.1M Tris-HCl at pH 8.0, 10% glycerol, 1% SDS) and boiled for 10 min. The protein concentration was measured by BCA method (Pierce, Rockford, IL, USA) and the lysates containing an equal amount of protein were separated by SDS-PAGE, transferred to PVDF membranes (Millipore, Darmstadt, Germany) for western blotting using the appropriate antibodies. The immunoreactive proteins were visualized using Clarity Western ECL substrate (Bio-Rad, Hercules, CA, USA), and light emission intensity was quantified using a LAS-3000 lumino-image analyzer equipped with Image Gauge version 2.3 software (Fuji, Tokyo, Japan). The antibodies used in this study were: anti-LXRβ rabbit monoclonal antibody (mAb) (Cell Signaling Technology, Danvers, MA USA; 13519), anti-β-actin mouse mAb (Sigma A5316), and anti-VHL rabbit polyclonal antibody (Cell Signaling Technology, 68547).

**siRNA transfection**

Cells were transiently transfected with a gene-specific siRNA (Life Technologies) or negative control siRNA (Qiagen) using Lipofectamine RNAi MAX reagent (Life Technologies). Lipofectamine RNAi MAX reagent (5 µL) and siRNA (100 pmol) were mixed in 500 µL Opti-MEM I Reduced Serum Medium (Life Technologies) on 6 well cell culture plate. After 10-20 minutes at room temperature, cells in complete growth medium without antibiotics were added into the plate and incubated at 37 ˚C in a humidified atmosphere of 5% CO_2_ for the indicated periods. The siRNA sequences used in this study were as follows: VHL-1 (5’-GCUUGUAUGUAAGGAGGUU-3’); VHL-2 (5’- GGAAUUGCAGCAUAUCGUU-3’); VHL-3 (5’- CCUGCACAUCAUGAGCCUU-3’).

# References

1) Zengerle, M., Chan, K., and Ciulli, A. (2015). Selective small molecule induced degradation of the BET bromodomain Protein BRD4. *ACS* *Chem*. *Biol*. 10, 1770-1777.

2) Ishoey, M., Chorn, S., Singh N., Jaeger, M. G., Brand, M., Paulk J., Bauer, S., Erb, M. A., Parapatics, K., Müller, A. C., Bennett, K. L., Ecker, G. F., Bradner, J. E., and Winter, G. E. (2018). Translation Termination Factor GSPT1 Is a Phenotypically Relevant Off-Target of Heterobifunctional Phthalimide Degraders. *ACS* *Chem*. *Biol*. 13, 533-560.

3) Olson, C. M., Jiang B., Erb, M. A., Liang Y., Doctor, Z. M., Zhang Z., Zhang, T., Kwiatkowski, N., Boukhali, M., Green, J. L., Haas, W., Nomanbhoy, T., Fischer, E. S., Young, R. A., Bradner, J. E., Winter, G. E., and Gray, N. S. (2018). Pharmacological perturbation of CDK9 using selective CDK9 inhibition or degradation. *Nat*. *Chem*. *Biol*. 14, 163-170.
